# Confirmatory spirometry for adults hospitalized with a diagnosis of asthma or chronic obstructive pulmonary disease exacerbation

**DOI:** 10.1186/1471-2466-12-73

**Published:** 2012-12-07

**Authors:** Valentin Prieto Centurion, Frank Huang, Edward T Naureckas, Carlos A Camargo Jr, Jeffrey Charbeneau, Min J Joo, Valerie G Press, Jerry A Krishnan

**Affiliations:** 1 University of Illinios at Chicago, Chicago, IL, USA; 2 University of Chicago Medicine, Chicago, IL, USA; 3 Massachusetts General Hospital, Harvard Medical School, Boston, MA, USA; 4 University of Illinois Hospital & Health Sciences System, Medical Center Administration Building, 914 S. Wood Street, MC 973, Chicago, IL, 60612, USA

**Keywords:** Asthma, COPD, Exacerbation, Hospitalization, Spirometry, Quality improvement

## Abstract

**Background:**

Objective measurement of airflow obstruction by spirometry is an essential part of the diagnosis of asthma or COPD. During exacerbations, the feasibility and utility of spirometry to confirm the diagnosis of asthma or chronic obstructive pulmonary disease (COPD) are unclear. Addressing these gaps in knowledge may help define the need for confirmatory testing in clinical care and quality improvement efforts. This study was designed to determine the feasibility of spirometry and to determine its utility to confirm the diagnosis in patients hospitalized with a physician diagnosis of asthma or COPD exacerbation.

**Methods:**

Multi-center study of four academic healthcare institutions. Spirometry was performed in 113 adults admitted to general medicine wards with a physician diagnosis of asthma or COPD exacerbation. Two board-certified pulmonologists evaluated the spirometry tracings to determine the proportion of patients able to produce adequate quality spirometry data. Findings were interpreted to evaluate the utility of spirometry to confirm the presence of obstructive lung disease, according to the 2005 European Respiratory Society/American Thoracic Society recommendations.

**Results:**

There was an almost perfect agreement for acceptability (κ = 0.92) and reproducibility (κ =0.93) of spirometry tracings. Three-quarters (73%) of the tests were interpreted by both pulmonologists as being of adequate quality. Of these adequate quality tests, 22% did not present objective evidence of obstructive lung disease. Obese patients (BMI ≥30 kg/m^2^) were more likely to produce spirometry tracings with no evidence of obstructive lung disease, compared to non-obese patients (33% vs. 8%, p = 0.007).

**Conclusions:**

Adequate quality spirometry can be obtained in most hospitalized adults with a physician diagnosis of asthma or COPD exacerbation. Confirmatory spirometry could be a useful tool to help reduce overdiagnosis of obstructive lung disease, especially among obese patients.

## Background

Exacerbations of asthma or chronic obstructive pulmonary disease (COPD), the most common obstructive lung diseases, account for more than one million hospitalizations and nearly six million hospital days each year in the US alone [1-4]. Readmission rates at 30 days, following hospitalization for asthma and COPD exacerbations, are approximately 10% and 20%, respectively [5-7]. Readmission rates at 90-days in patients with COPD exacerbations are estimated to be about 35% [8]. In-hospital mortality for patients admitted with asthma or COPD exacerbations ranges from 0.2% to 38%; higher mortality rates correspond to populations with a greater acuity of illness, including those requiring mechanical ventilation [2,7-9]. The economic burden from these hospitalizations and re-admissions is enormous; annual direct costs are estimated to be $16 billion, representing more than 30% of the total medical care costs for these two conditions [3].

Studies performed using International Classification of Diseases, ninth revision, (ICD-9) billing codes or physician-documented diagnosis to identify the study population, indicate that up to 50% of patients hospitalized with asthma or COPD exacerbations do not receive guideline recommended care [10,11]. In a previous study using chart abstraction, we found that relying on ICD-9 billing codes may lead to overdiagnosis of COPD exacerbations in up to 25% of patients, potentially because confirmatory testing (e.g. spirometry) to document obstructive lung disease is rarely performed [12]. To our knowledge, similar data on asthma exacerbations is not available. Objective measurement of expiratory airflow obstruction is considered essential to the diagnosis of asthma and COPD, as other diseases can present with similar symptoms. A recently completed audit of patients hospitalized for COPD exacerbations found substantial variations in care, with spirometry prior to hospital admissions available in only about three-quarters of patients [8]. While confirmatory spirometry is recommended by the European Respiratory Society/American Thoracic Society guidelines to establish a diagnosis of asthma or COPD, is not routinely performed during hospitalizations for exacerbations, due to concerns about its feasibility (e.g., inadequate test quality) and a lack of data supporting its utility.

There is a paucity of data about the feasibility of measuring lung function in *hospitalized* patients suspected of having an asthma or COPD exacerbation. A recent single-hospital study by Rea and colleagues [13] found that spirometry, performed upon hospital discharge, can serve as a baseline against which post-discharge measurements can be compared. However, we are not aware of studies that have specifically examined the quality of spirometry tests obtained early in the course of hospitalizations for patients with COPD or asthma exacerbations and their utility in confirming the presence of obstructive lung disease.

Rea and colleagues also showed that approximately 16% of patients hospitalized with COPD exacerbations did not meet the GOLD criteria for COPD by spirometry on hospital discharge [13]. Data about the prevalence of patients with a physician diagnosis of an asthma exacerbation but in whom spirometry fails to confirm obstructive lung disease (i.e., overdiagnosis) are lacking.

To address these gaps in knowledge, we conducted a multi-center study in adults hospitalized with a physician diagnosis of asthma or COPD exacerbation to: a) evaluate the quality of spirometry tracings; and b) assess the utility of confirmatory spirometry for the presence of obstructive lung disease in patients hospitalized with a physician diagnosis of asthma or COPD exacerbation. The findings reported in this study may help determine the need for confirmatory testing in clinical care setting, or as part of quality improvement efforts, such as pay-for-performance, in adults hospitalized with a physician diagnosis of asthma or COPD exacerbation.

## Methods

### Patient population

As part of several hospital-based studies [14,15], we screened admission logs to identify adults admitted for asthma or COPD exacerbations at four university-affiliated medical centers (The Johns Hopkins Hospital, Johns Hopkins Bayview Medical Center, The University of Chicago Medical Center, and Mercy Hospital and Medical Center). The general medicine treating physician of each potential participant was contacted for verbal assent to approach their patient, using standardized text, and to confirm the diagnosis of asthma or COPD exacerbation. Since the participants received standard care while in the hospital, a physician diagnosis of asthma or COPD exacerbation was sufficient. Written informed consent was obtained from patients who met all inclusion criteria (age ≥18 years, admitted to the general ward, and physician diagnosis of asthma or COPD exacerbation). Patients with additional respiratory diagnosis (e.g., sarcoidosis), too ill to provide informed consent according to the treating physician, or admitted to the intensive care unit at the time of screening were excluded. Demographic information (date of birth, gender, self-reported height, self-reported weight) was collected from patients at the time spirometry was performed. Medical records were reviewed to collect data on the date of hospital admission and discharge. The study was approved by the Institutional Review Board at each medical center (University of Chicago Medical Center protocol numbers 15729A, 14831A, John Hopkins Hospital and John Hopkins Bayview Medical Center protocol numbers 03-08-19-02, 03-08-10-02, no protocol number provided by Mercy Hospital and Medical Center).

### Study procedures

As part of the study procedures, admission logs were scanned daily to identify potential study participants. Spirometry is rarely performed on hospital admission as part of routine clinical care. Thus, for this study, spirometry was performed as early as possible during hospitalization without interfering with patient care (e.g., treatments, other tests, evaluations being performed by the clinical team). Study staff administered 2 puff of inhaled albuterol and conducted post-bronchodilator spirometry tests at the bedside. Spirometry tests were performed using a Koko spirometer (KoKo®; Pulmonary Data Services Instrumentation; Louisville, CO) while participants were seated in their hospital room. Spirometry with flow volume loops were obtained using European Respiratory Society/American Thoracic Society (ERS/ATS) recommendations; each participant completed up to eight efforts to measure the FEV_1_ and FVC [16].

### Assessment of quality of spirometry tracings

Two board-certified pulmonologists independently rated spirometry tracings according to the ERS/ATS criteria [17]. To be considered of adequate quality, spirometry tracings had to satisfy the criteria for both acceptability and reproducibility. A spirometry tracing was considered acceptable if it showed at least three efforts meeting criteria for an acceptable beginning of test (back-extrapolated volume [BEV] <150ml or <5% of forced vital capacity [FVC], whichever is greater), middle of test (no artifacts [e.g., glottis closure, cough or hesitation]), and end of test (exhalation of at least 6 seconds or a plateau before 6 seconds). A test was considered reproducible if the difference between the two highest forced expired volumes in the first second (FEV_1_) and FVC values were both <150ml (or <100ml if FVC <1L). A spirometry test had to fulfill the criteria for acceptability and reproducibility by both raters to be considered adequate quality.

### Assessment of utility of confirmatory spirometry

The utility of confirmatory spirometry was determined by assessing the percentage and demographic characteristics of patients without evidence of airflow obstruction by spirometry. Obstructive lung disease was defined as FEV_1_/FVC < lower limit of normal (LLN); the LLNs were calculated using NHANES III-predicted equations [18].

### Statistical analysis

Descriptive statistics employed proportions. Medians and interquartile range (IQR) were used to describe the days from hospital admission to spirometry testing. We calculated the kappa (κ) statistic to evaluate agreement between raters regarding acceptability and reproducibility of spirometry tracings [19]. Body-mass index (BMI) was calculated and categorized according to the WHO criteria [20]. Bivariate analyses employed a χ^2^, Fisher’s exact, or Wilcoxon rank-sum tests, where appropriate. All reported p-values are two sided, and p-values of <0.05 were considered statistically significant. Analyses were performed using STATA software package, release 10.0 (Stata Corp Inc, College Station, Texas).

## Results

### Patient characteristics

Of the 113 participants in the study, 68% were female, 63% were between the ages of 35 and 64, 56% were obese and 59% were admitted with a diagnosis of asthma exacerbation. Participants with a diagnosis of asthma exacerbation were younger and had higher BMIs (e.g., 61% vs. 48% had BMI ≥30 kg/m^2^) than those with a diagnosis of COPD exacerbation (Table 1). The median (IQR) time from admission to spirometry testing was 1 day (1 to 2 days).

**Table 1:** Patient characteristics*

**Characteristic**	**All n = 113**	**Physician diagnosis**		**p-value**
		**Asthma exacerbation n = 67 (59%)**	**COPD exacerbation n = 46 (41%)**	
**Age, years**				
<35	20 (18%)	18 (27%)	2 (4%)	<0.0001
35-64	71 (63%)	44 (66%)	27 (59%)	
≥65	22 (19%)	5 (7%)	17 (37%)	
**Female**	77 (68%)	48 (72%)	29 (63%)	0.41
**BMI, kg/m**^**2**^				
<18.5 (Underweight)	7 (6%)	0 (0%)	7 (15%)	0.009
18.5-24.9 (Normal)	22 (19%)	11 (16%)	11 (24%)	
25-29.9 (Overweight)	21 (19%)	15 (22%)	6 (13%)	
≥30 (Obese)	63 (56%)	41 (61%)	22 (48%)	
**Median days from hospital admission spirometry testing (IQR)**	1 (1 to 2)	1 (1 to 2)	1 (1 to 2)	0.77

Values above represent n (column %), unless otherwise specified.

Abbreviations: IQR, interquartile range.

* Due to rounding, percentages may not add to 100%.

### Spirometry quality

Overall, nearly three-quarters of spirometry tests (73%) were graded as being of adequate quality, with a similar proportion in patients with a diagnosis of asthma and COPD exacerbation (Table 2). There were discordant interpretations by the two raters regarding the acceptability of only 3 (2%) spirometry tracings; there were no discordant interpretations regarding reproducibility among tracings graded as being acceptable by both raters. There was almost perfect agreement for acceptability (κ = 0.92) and reproducibility (κ =0.93).

**Table 2:** Characteristics of patients according to spirometry quality*

**Characteristic**	**Adequate quality**	**Inadequate quality**	**p-value**
	**n = 83 (73%)**	**n = 30 † (27%)**	
**Physician diagnosis**			
Asthma exacerbation	47 (78%)	20 (22%)	0.23
COPD exacerbation	36 (70%)	10 (30%)	
**Age, years**			
<35	14 (70%)	6 (30%)	0.91
35-64	53 (75%)	18 (25%)	
≥65	16 (73%)	6 (27%)	
**Female**	57 (73%)	21 (27%)	0.50
**BMI, kg/m**^**2**^			
<18.5 (Underweight)	5 (71%)	2 (29%)	0.82
18.5-24.9 (Normal)	16 (73%)	6 (27%)	
25-29.9 (Overweight)	17 (81%)	4 (19%)	
≥30 (Obese)	45 (71%)	18 (29%)	
**Median days from hospital admission to spirometry testing (IQR)**	1 (1 to 2)	1 (0 to 2)	0.06

Values above represent n (row %), unless otherwise specified.

Abbreviations: *IQR* Interquartile range.

* Due to rounding, percentages may not add to 100%.

† Includes spirometry tracings in which one (n = 25, 22%) or both (n = 22, 19%) raters graded the effort as not acceptable or not reproducible based on ERS/ATS criteria.

Among tracings interpreted as not being acceptable by at least one rater (n = 25/113 for rater 1; n = 22/113 for rater 2), the most common reason was failure to meet end of test criteria (20/25 for rater 1; 17/22 for rater 2). Tests also failed to meet start of test criteria (11/25 for rater 1; 11/22 for rater 2) or middle of test criteria (14/25 for rater 1; 11/22 for rater 2). Reasons for which tests failed to meet acceptability criteria did not vary depending on age, gender, physician diagnosis or BMI (Additional file 1: Table S1).

There was a trend suggesting that tracings rated as not adequate quality were associated with a shorter interval between hospital admission and performance of spirometry (median [IQR] days 1 [0–1] for not adequate quality tests vs. 1 [0–2] for adequate quality, p = 0.06). None of the other patient characteristics we examined were associated with adequate vs. not adequate spirometry tracings (p-values >0.2).

### Spirometry interpretation and assessment of utility

Among the 83 tests that were considered to be adequate quality by both raters, obstructive lung disease was confirmed in only four of five tests (78%) (Table 3). In those with confirmed obstructive lung disease, the mean (SD) % predicted FEV_1_ and FEV_1_/FVC were 43% (16%) and 56% (10%), respectively. In those with a diagnosis of asthma exacerbation, obstructive lung disease was confirmed in 83% versus 72% with a diagnosis of COPD exacerbation (p = 0.29). Thus, approximately 1 in 5 patients did not have spirometric evidence of obstructive lung disease. Participants without evidence of obstructive lung disease were significantly more likely to have higher BMIs (p = 0.009). Obese participants (i.e., BMI ≥30 kg/m^2^) were four times more likely than non-obese participants to be misclassified as having airflow obstruction (33 vs. 8%, p = 0.007). None of the other patient characteristics we examined were associated with a lack of evidence of obstructive lung disease. The number of days between hospital admission and spirometry testing was also not associated with a lack of obstructive lung disease.

**Table 3:** Characteristics of patients with adequate quality spirometry (n = 83) with and without obstructive lung disease *

**Characteristics**	**Obstructive lung disease**	**No obstructive lung disease**	**p-value**
	**n = 65 (78%)**	**n = 18 (22%)**	
**Clinical diagnosis**			
Asthma exacerbation	39 (83%)	8 (17%)	0.29
COPD exacerbation	26 (72%)	10 (28%)	
**Age, years**			
<35	11 (79%)	3 (21%)	0.55
35-64	43 (81%)	10 (19%)	
≥65	11 (69%)	5 (31%)	
**Female**	42 (72%)	14 (25%)	0.40
**BMI, kg/m**^**2**^			
<18.5 (Underweight)	4 (80%)	1 (20%)	0.009
18.5-24.9 (Normal)	16 (100%)	0 (0%)	
25-29.9 (Overweight)	15 (88%)	2 (12%)	
≥30 (Obese)	30 (67%)	15 (33%)	
**Median days from hospital admission to spirometry testing (IQR)**	1 (1 to 2)	1 (1 to 2)	0.30

Values represent n (row %), unless otherwise noted. Abbreviations: *IQR* Interquartile range.

* Only in patients for which both raters reported acceptable and reproducible tracings.

Spirometry and flow volume loop configurations suggested alternate abnormalities, including variable extrathoracic airflow obstruction and restrictive lung disease among tests that did not meet criteria for obstructive lung disease.

## Discussion

In this study, we demonstrated that adequate quality spirometry can be obtained in three-quarters of hospitalized patients with a physician diagnosis of asthma or COPD exacerbation. Spirometry confirmed obstructive lung disease in 78% of participants with adequate quality tests; in other words, about 1 in 5 participants with a physician diagnosis of asthma or COPD exacerbation did not meet the diagnostic criteria by spirometry. Overdiagnosis was about four times more likely in obese than in non-obese patients.

Our finding demonstrates that adequate quality spirometry can be obtained early in the hospital course in most patients with asthma or COPD exacerbations. This finding expands previous work by Rea and colleagues [13], who showed that patients hospitalized with COPD exacerbations were able to perform spirometry on the day of discharge. None of the participant characteristics we examined were different between those who were able to produce adequate vs. not adequate quality spirometry, except for a trend between the number of days between hospital admission and spirometry testing. We found a trend suggesting that a shorter number of days between hospital admission and spirometry testing may be associated with the inability to produce adequate spirometry. We suspect that the length of hospital stay before spirometry testing is linked to the acuity of illness (i.e. sicker patients were unable to perform spirometry). Therefore, an important limitation to our study is that patients who were too ill to provide informed consent and those who were admitted to the intensive care unit were excluded; such patients may be even more likely to not perform adequate quality spirometry tests.

Recent studies of spirometry in the primary care setting have shown that up to 50% of patients with a physician diagnosis of COPD did not meet the criteria for the diagnosis by spirometry [21-23]. Similarly, a recent study showed that more than 30% of patients with a physician diagnosis of stable asthma did not meet the criteria for the diagnosis when tested with a combination of spirometry and methacholine challenge test [24]. To our knowledge, the present report is the first study to evaluate the frequency of misclassification among hospitalized patients with a physician diagnosis of asthma exacerbation.

Additionally, we found that the lack of evidence for obstructive lung disease was four times more common in obese vs. non-obese participants. Our findings in hospitalized patients are consistent with results observed in a study of outpatients in a primary care setting that identified higher rates of misclassification for COPD in overweight or obese patients [22]. Similarly, previous studies have suggested that overweight or obese patients may be over-diagnosed as having asthma. For example, one study reported that nearly 1 in 3 overweight or obese subjects diagnosed with asthma do not actually have airway hyperresponsiveness [25]. Other studies, including some in obese patients, found that medical history and physical examination findings may not be sufficiently reliable to diagnose obstructive lung disease [26,27]. Other data suggest that vocal cord dysfunction may mimic an asthma exacerbation [28], which may help explain why some spirometry tracings had evidence of variable extrathoracic airflow obstruction. In our study, we found that some patients hospitalized with a diagnosis of asthma or COPD exacerbation presented spirometry tracings suggestive of restrictive (not obstructive) lung disease. Thus, together with previously published evidence, our findings suggest that a range of conditions may be contributing to respiratory symptoms diagnosed as asthma or COPD exacerbations.

This multicenter study had multiple strengths. First, spirometry was performed on average within 1 day of hospitalization, decreasing the likelihood that participants were tested after the resolution of the exacerbation. Second, we prospectively identified patients with a physician diagnosis of asthma or COPD exacerbation and in whom the treating physician did not suspect other respiratory conditions, rather than relying on chart abstraction or billing codes. Lastly, we employed standardized spirometry procedures using ERS/ATS guidelines, minimizing any variations in the procedure or interpretation.

There were also several potential limitations to our study. We relied on spirometry to diagnose obstructive lung disease and may have missed mild air trapping or increased peripheral airway resistance that would require the use of special diagnostic tests, such as body plethysmography or impulse oscillometry. However, ERS/ATS guidelines for the diagnosis of asthma or COPD are based on spirometry and do not require the use of such tests. Also, as participants were enrolled with respiratory symptoms requiring hospitalization, the likelihood of milder forms of airway disease requiring more sensitive tests would be very low. We relied on self-reported height and weight to determine the predicted spirometry results, which may not be as reliable as objective measurement of height and weight. We also enrolled a modest number of participants (n = 113) in four academic medical centers and we do not know if these results would be generalizable to other healthcare institutions (e.g., community hospitals). However, our results highlight the need for larger, multi-center studies to further evaluate the overdiagnosis of asthma and COPD exacerbations in hospitalized patients. Last, we only had limited data about patient characteristics. Additional information on the exacerbation severity and cognitive ability of the participants could have helped identify other characteristics associated with the inability to obtain adequate quality tests. We did not collect data regarding the comorbid conditions in patients without evidence of obstructive lung disease by spirometry (e.g., heart failure); this information may have offered clues into alternative diagnoses. Further, it is possible that the high proportion of obese patients without evidence of obstructive lung disease is due to residual confounding factors, such as respiratory muscle weakness, that were not measured in our study. Findings in this report can help to inform the design of larger multi-center, longitudinal studies that include community hospitals to assess differences in accuracy across institutions and within subgroups of patients.

## Conclusion

The study findings have several implications. First, the relatively high frequency of adequate quality spirometry tests (about three-quarters of patients tested) should be encouraging to clinicians who may want to use spirometry in the inpatient setting to confirm the diagnosis of asthma or COPD exacerbations. Second, the high rates of patients who did not meet the asthma or COPD diagnostic criteria by spirometry (about 20%), which were even higher among obese patients (33%). Given these findings, we recommend that clinicians routinely obtain spirometry in hospitalized patients suspected of having an asthma or a COPD exacerbation.

In conclusion, we found that adequate quality spirometry can be obtained in most patients hospitalized with exacerbations of asthma or COPD. Clinical practice and quality improvement efforts that include spirometry for confirmation of obstructive lung disease may help to reduce the risk of overdiagnosis, which could lead to inappropriate care in this population.

## Abbreviations

ATS: American Thoracic Society; BEV: Back extrapolated volume; BMI: Body-mass index; CMS: Centers for Medicare and Medicaid Services; COPD: Chronic obstructive pulmonary disease; ERS: European Respiratory Society; FEV_1_: Forced expiratory volume in 1 second; FVC: Forced vital capacity; GOLD: Global Initiative for Chronic Obstructive Lung Disease; ICD-9: International Classification of Diseases, Ninth Revision; IQR: Interquartile range (25th and 75th percentile); LLN: Lower limit of normal; NHANES: National Health and Nutrition Examination Survey.

## Competing interests

The authors declare that they do not have competing interests.

## Authors’ contributions

Dr VPC had full access to all of the data in the study and takes responsibility for the integrity of the data and the accuracy of the data analysis. Drs VPC, FH, EN and JK contributed to study design, data analysis and interpretation, and preparation of the manuscript. Drs CC, MJ and VGP contributed to the data interpretation, and preparation of this manuscript. Mr JC contributed to the data analysis and interpretation, and preparation of the manuscript. All authors read and approved the final manuscript.

## Sources of funding

Funding for this study was received from the Agency for Healthcare Research and Quality (R13 HS017894) and National Heart, Lung, and Blood Institute (HL1011618). The sponsors had no role in the design of the study, collection and analysis of the data, or in the preparation of the manuscript.

## Pre-publication history

The pre-publication history for this paper can be accessed here:

http://www.biomedcentral.com/1471-2466/12/73/prepub

## Supplementary Material

Additional file 1 **Table S1.** Characteristics of patients with acceptable and not acceptable spirometry. Click here for file
