# Supplementary material for: Confirmatory spirometry for adults hospitalized with a diagnosis of asthma or chronic obstructive pulmonary disease exacerbation
Source: BMC Pulm Med. 2012 Dec 7;12:73. doi: 10.1186/1471-2466-12-73 (PMC3538708; doi:10.1186/1471-2466-12-73)
Supplement: Additional file 1 — Table S1. Characteristics of patients with acceptable and not acceptable spirometry. [file 1471-2466-12-73-S1.doc]

**APPENDIX**

**Table A.1.** Characteristics of patients with acceptable and not acceptable spirometry

| **Characteristics** | **Acceptable** | **Not acceptable** | **p-value** |
| --- | --- | --- | --- |
|  | n=88 (78%) | n=25 (22%) |  |
| **Clinical diagnosis** | | | |
| Asthma exacerbation | 52 (78%) | 15 (22%) | 0.93 |
| COPD exacerbation | 36 (78%) | 10 (22%) |  |
|  |  |  |  |
| **Age, years** |  |  |  |
| <35 | 15 (75%) | 5 (25%) | 0.73 |
| 35-64 | 57 (80%) | 14 (20%) |  |
| ≥65 | 16 (73%) | 6 (27%) |  |
|  |  |  |  |
| **Female** | 59 (77%) | 18 (23%) | 0.64 |
|  |  |  |  |
| **BMI, kg/m^2^** |  |  |  |
| <18.5 (Underweight) | 5 (71%) | 2 (29%) | 0.8 |
| 18.5-24.9 (Normal) | 17 (77%) | 5 (23%) |  |
| 25-29.9 (Overweight) | 18 (86%) | 3 (14%) |  |
| ≥30 (Obese) | 48 (76%) | 15 (24%) |  |
|  |  |  |  |
| **Median days from hospital admission to spirometry testing (IQR)** | 1 (1 to 2) | 1 (1 to 2) | 0.36 |

Values represent n (row %), unless otherwise noted. Abbreviations: IQR, interquartile range.
